# Supplementary material for: Tracing the emergence of domesticated grapevine in Italy
Source: PLoS One. 2025 Apr 23;20(4):e0321653. doi: 10.1371/journal.pone.0321653 (PMC12017526; doi:10.1371/journal.pone.0321653)

Dorsal view (VD)

Cumulative harmonic power %

H1	H2	H3	H4	H5	H6	H7	H8	H9	H10	H11
59.4	82.6	92.0	94.8	98.3	99.0	99.6	99.7	99.9	99.9	100.0

Calibrate reconstruction EFT based on a randomly selected pip of the «Carignan» variety

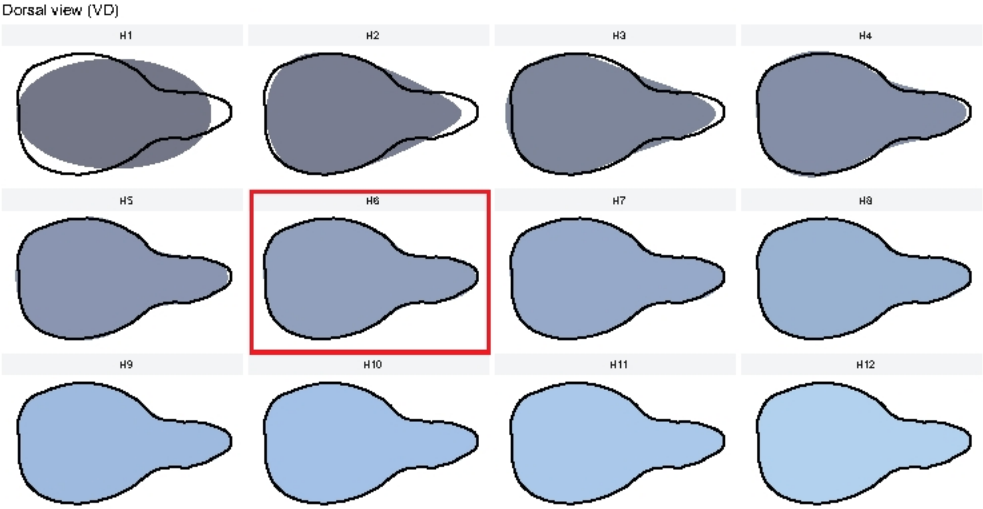

Lateral view (VL)

Cumulative harmonic power %

H1	H2	H3	H4	H5	H6	H7	H8	H9	H10	H11
38.7	88.0	91.5	97.5	98.9	99.3	99.6	99.7	99.9	99.9	100.0

Calibrate reconstruction EFT based on a randomly selected pip of the «Carignan» variety

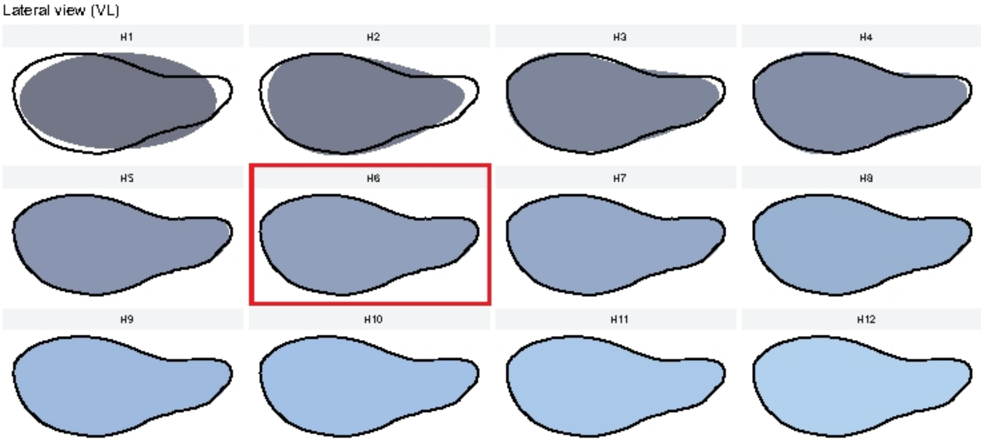

Supplement: S1 Fig — For each view, a) cumulated amount of information brought by the first 12 harmonics based on a set of 100 randomly selected modern pips of the reference collection, b) reconstruction of pip shape for a randomly selected modern pip of the “Carignan” cultivar. (PDF) [file pone.0321653.s003.pdf]
