# Supplementary material for: Tracing the emergence of domesticated grapevine in Italy
Source: PLoS One. 2025 Apr 23;20(4):e0321653. doi: 10.1371/journal.pone.0321653 (PMC12017526; doi:10.1371/journal.pone.0321653)
Supplement: Attachment — Submitted filename: Review Tracing the Grapevine in Italy.docx [file pone.0321653.s007.docx]

Peer review report for "Tracing the Emergence of Domesticated Grapevine in Italy"

- What are the main claims of the paper and how significant are they for the discipline?

The paper’s focus is the question of the appearance of the domesticated form of grapes in Italy. This is the continuation of the research – see REF # 17-19. In a way, the presented research elaborates the method, and the data presented in Breglia, F., Bouby, L., Wales, N. *et al.* Disentangling the origins of viticulture in the western Mediterranean. *Sci Rep* **13**, 17284 (2023). <https://doi.org/10.1038/s41598-023-44445-4>

- Are the claims properly placed in the context of the previous literature? Have the authors treated the literature fairly?

The reference list is incorrect. Page 2 [7] – Mangafa is not the source for the size and shape of wild vs cultivated pips. [13] – Dong et al. is the source for the two simultaneous domestication events 11,000 years ago in W Asia and Caucasus, which led to different routes and timing of dispersal and further introgressions of the primary cultivars. The reference [16] is dated to 2021, while [13] to 2023 – so the style of the sentence “However, the hypothesis of a second, independent, grape domestication event outside the original primary domestication center is still [sic! - reviewer] debated” - is confusing and misleading.

ETC. It must be updated and fixed.

Page 2—Connection of the spread of viticulture with “emergence of complex societies”—please add more here for those who are not familiar with the development of societies in Italy. Add that in Italy, the first evidence of a hierarchical society was during the MB.

When citing only one single work in a sentence, such as “different research has employed pip outline analysis study grape subspecies [20] – the chosen reference should be a review, or please add “e.g.” before the single chosen work.

- Do the data and analyses fully support the claims? If not, what other evidence is required?

The method applied in the research, Fourier transforms, is widely used for morphometric identification of plants, and grape pips. It is a strong method for machine learning identification. Unfortunately, the method cannot be used for manual identification, but this will be, hopefully, addressed later.

Nevertheless, it is important to add here in this paper a figure/ table to visualize the choice of the “six first harmonics, that were utilized here (although it is a continuation of the previously published method – ref 20 and 30. Accordingly, the data of the measurements – the raw data for statistics, should be presented. Now, it is presented in figure 4, where individual measurements are unclear.

Next, the “48 EFT coefficients” – should be presented in the paper.

As for the results, means and covariances for the wild and domesticated types, as well, means and covariances for each period should be presented in the paper. This can be estimated from the training set. In this case, the data will help the readers to follow the authors and will allow us to evaluate the results, and to apply the results in future studies of other grape populations. Presenting the data will enlarge our understanding of the changes in the grape pip’s morphology with time and domestication.

Each method has its cautions and limitations, so it is critical to address this issue, and to discuss the pros and contra of applying the method on the results.

- *PLOS ONE* encourages authors to publish detailed protocols and algorithms as supporting information online. Do any particular methods used in the manuscript warrant such treatment? If a protocol is already provided, for example for a randomized controlled trial, are there any important deviations from it? If so, have the authors explained adequately why the deviations occurred?

The paper does not present the raw data, so it is not possible to follow their protocol, nor to evaluate the data significance.

- If the paper is considered unsuitable for publication in its present form, does the study itself show sufficient potential that the authors should be encouraged to resubmit a revised version?

The protocol must be presented (what are the vectors, and why they were chosen); and the data must be presented (mean, covar). Now it is unclear.

In sum, for this article to be published, a clear protocol and the resulting data should be presented.
