# Supplementary material for: Tracing the emergence of domesticated grapevine in Italy
Source: PLoS One. 2025 Apr 23;20(4):e0321653. doi: 10.1371/journal.pone.0321653 (PMC12017526; doi:10.1371/journal.pone.0321653)
Supplement: Attachment — Submitted filename: Response to Reviewers.docx [file pone.0321653.s008.docx]

**Reference** PONE-D-24-32533 *Tracing the Emergence of Domesticated Grapevine in Italy*

PLOS ONE

Dear editor,

We are grateful for the helpful feedback by the reviewers that helped us to improve the quality of the manuscript. We carefully responded to all points.

**Response to the academic editor:**

**1.** Please ensure that your manuscript meets, including those for file naming.

**Response 1**

We followed the PLOS ONE's style requirements.

**2.** In your manuscript, please provide additional information regarding the specimens used in your study. Ensure that you have reported human remain specimen numbers and complete repository information, including museum name and geographic location.

If permits were required, please ensure that you have provided details for all permits that were obtained, including the full name of the issuing authority, and add the following statement:

'All necessary permits were obtained for the described study, which complied with all relevant regulations.'

If no permits were required, please include the following statement:

'No permits were required for the described study, which complied with all relevant regulations.'

**Response 2**

We added the following statement: “All necessary permits were obtained for the described study, which complied with all relevant regulations.”

**3.** We note that the grant information you provided in the ‘Funding Information’ and ‘Financial Disclosure’ sections do not match.

When you resubmit, please ensure that you provide the correct grant numbers for the awards you received for your study in the ‘Funding Information’ section.

We note that you have provided funding information that is not currently declared in your Funding Statement. However, funding information should not appear in the Acknowledgments section or other areas of your manuscript. We will only publish funding information present in the Funding Statement section of the online submission form.  Please remove any funding-related text from the manuscript and let us know how you would like to update your Funding Statement. Currently, your Funding Statement reads as follows:  M.U. received funding from the European Union Horizon 2020 research and innovation programme under the Marie Skłodowska-Curie grant agreement (No 101019563 –VITALY).

Please include your amended statements within your cover letter; we will change the online submission form on your behalf.

**Response 3**

We removed the funding information from the acknowledgments section; we would like the following statement to be included: “This research has received funding from the European Union’s Horizon 2020 research and innovation programme under the Marie Sklodowska-Curie grant agreement No 101019563. L. Bouby and S. Ivorra were supported by the ANR MICA project (grat agreement ANR-22-CE27-0026).”

**5.** We note that you have indicated that there are restrictions to data sharing for this study. PLOS only allows data to be available upon request if there are legal or ethical restrictions on sharing data publicly. For more information on unacceptable data access restrictions, please see http://journals.plos.org/plosone/s/data-availability#loc-unacceptable-data-access-restrictions.

Before we proceed with your manuscript, please address the following prompts:

a) If there are ethical or legal restrictions on sharing a de-identified data set, please explain them in detail (e.g., data contain potentially identifying or sensitive patient information, data are owned by a third-party organization, etc.) and who has imposed them (e.g., a Research Ethics Committee or Institutional Review Board, etc.). Please also provide contact information for a data access committee, ethics committee, or other institutional body to which data requests may be sent.

b) If there are no restrictions, please upload the minimal anonymized data set necessary to replicate your study findings to a stable, public repository and provide us with the relevant URLs, DOIs, or accession numbers. For a list of recommended repositories, please see https://journals.plos.org/plosone/s/recommended-repositories. You also have the option of uploading the data as Supporting Information files, but we would recommend depositing data directly to a data repository if possible.

We will update your Data Availability statement on your behalf to reflect the information you provide.

**Response 5**

All data are now publicly available (see further answers).

**6.** We note that Figures 1 and 3 in your submission contain [map/satellite] images which may be copyrighted. All PLOS content is published under the Creative Commons Attribution License (CC BY 4.0), which means that the manuscript, images, and Supporting Information files will be freely available online, and any third party is permitted to access, download, copy, distribute, and use these materials in any way, even commercially, with proper attribution. For these reasons, we cannot publish previously copyrighted maps or satellite images created using proprietary data, such as Google software (Google Maps, Street View, and Earth). For more information, see our copyright guidelines: http://journals.plos.org/plosone/s/licenses-and-copyright.
 
We require you to either (1) present written permission from the copyright holder to publish these figures specifically under the CC BY 4.0 license, or (2) remove the figures from your submission:

a. You may seek permission from the original copyright holder of Figures 1 and 3 to publish the content specifically under the CC BY 4.0 license.

We recommend that you contact the original copyright holder with the Content Permission Form (http://journals.plos.org/plosone/s/file?id=7c09/content-permission-form.pdf) and the following text:
“I request permission for the open-access journal PLOS ONE to publish XXX under the Creative Commons Attribution License (CCAL) CC BY 4.0 (http://creativecommons.org/licenses/by/4.0/). Please be aware that this license allows unrestricted use and distribution, even commercially, by third parties. Please reply and provide explicit written permission to publish XXX under a CC BY license and complete the attached form.” Please upload the completed Content Permission Form or other proof of granted permissions as an ""Other"" file with your submission.

In the figure caption of the copyrighted figure, please include the following text: “Reprinted from [ref] under a CC BY license, with permission from [name of publisher], original copyright [original copyright year].”
 
b. If you are unable to obtain permission from the original copyright holder to publish these figures under the CC BY 4.0 license or if the copyright holder’s requirements are incompatible with the CC BY 4.0 license, please either i) remove the figure or ii) supply a replacement figure that complies with the CC BY 4.0 license. Please check copyright information on all replacement figures and update the figure caption with source information. If applicable, please specify in the figure caption text when a figure is similar but not identical to the original image and is therefore for illustrative purposes only.

**Response 6**

I have revised Figures 1 and 3. The current figures are the result of my personal work in vector graphics.

**7.** Please include captions for your Supporting Information files at the end of your manuscript, and update any in-text citations to match accordingly.

**Response 7**

We included the captions for our Supporting Information files at the end of our manuscript.

**Reviewer's Responses to Questions**

**Reviewers' comments:**

**Reviewer #1:** The authors reported the morphometrics analysis of several waterlogged archaeological grape pips collected in Italian archaeological sites and covering 7000 years of history. Using geometric morphometrics and linear discriminant analyses, they suggested insights into the evolution of grapevine cultivation in Italy and the transition from wild to domesticated grapevines.
The manuscript reports interesting and original results and provides a potential contribution to understanding the diffusion and evolution of viticulture in Italy. These data should be supported in the future by genetic analyses that could suggest links with modern varieties cultivated in Italy and Southern Europe.
I suggest reporting the numerical results of the pips classification (wild, domesticate, non allocated) in the supplementary table SM2 next to the discovery sites. In this way the first part of the results (lines 229-260) could be lightened by many numerical data to make it less "boring" and similar to a list.

**Response**

Following your suggestion, we have included all the seed classification results in the supporting file SM2, now renamed “S2A Table and S2B Table”. We also revised part of the manuscript by removing numerical data from the text.

In addition, to meet the needs of open research, I would suggest that the authors report the raw data of all the measurements conducted on all the pips (in the supplementary materials or in some public repository), both the modern ones used to create the reference database and the archaeological ones.

**Response**

We reported the raw data of all the measurements conducted on archaeological pips in the supplementary file “S3 Table” The full dataset related to the modern reference collection was already made available in Bonhomme et al. 2021.

**Reviewer #2:**

**1.** The method applied in the research, Fourier transforms, is widely used for morphometric identification of plants, and grape pips. It is important to add here in this paper a figure/ table to visualize the choice of the “six first harmonics, that were utilized here (although it is a continuation of the previously published method – Ref #20 and #30. Accordingly, the data of the measurements – the raw data for statistics, should be presented. Now, it is presented in figure 4, where individual measurements are unclear.

**Response**

All data related to the modern reference collection and archaeological pips are now fully available (see answer to previous comment). We have added as supplementary data a figure showing a) for each view, the cumulated amount of information brought by each of the first 12 harmonics for a set of 100 randomly selected modern seeds of the reference collection, and b) a reconstruction of seed shape a randomly selected pip of the “Carignan” modern cultivar based on the information brought by each harmonic (S1_fig.pdf).

Next, the “48 EFT coefficients” – should be presented in the paper. As for the results, means and covariances for the wild and domesticated types, as well, means and covariances for each period should be presented in the paper. This can be estimated from the training set. In this case, the data will help the readers to follow the authors and will allow us to evaluate the results, and to apply the results in future studies of other grape populations. Presenting the data will enlarge our understanding of the changes in the grape pip’s morphology with time and domestication. Each method has its cautions and limitations, so it is critical to address this issue, and to discuss the pros and contra of applying the method on the results.

**Response**

We added a table providing mean and variance values for the 48 EFT coefficients and Pip length, for both the domesticated and wild types and for each period (S4 Table).

**2.** The reference list is incorrect. Page 2 [7] – Mangafa is not the source for the size and shape of wild vs cultivated pips. [13] – Dong et al. is the source for the two simultaneous domestication events 11,000 years ago in W Asia and Caucasus, which led to different routes and timing of dispersal and further introgressions of the primary cultivars. The reference [16] is dated to 2021, while [13] to 2023 – so the style of the sentence “However, the hypothesis of a second, independent, grape domestication event outside the original primary domestication center is still [sic! - reviewer] debated” - is confusing and misleading.

**Response 2.**

Thank you for the suggestion. We have replaced the reference and revised the sentence to make it clearer.

Page 2—Connection of the spread of viticulture with “emergence of complex societies”—please add more here for those who are not familiar with the development of societies in Italy. Add that in Italy, the first evidence of a hierarchical society was during the MB.

**Response**

We have added a sentence to indicate the emergence of complex societies in Italy.

When citing only one single work in a sentence, such as “different research has employed pip outline analysis study grape subspecies [20] – the chosen reference should be a review, or please add “e.g.” before the single chosen work.

**Response**

We have added the relevant bibliographic references to the works that used the pip outline analysis study grape subspecies.

**Peer review report for “Tracing the Emergence of Domesticated Grapevine in Italy”**

What are the main claims of the paper and how significant are they for the discipline?

The paper’s focus is the question of the appearance of the domesticated form of grapes in Italy. This is the continuation of the research – see REF # 17-19. In a way, the presented research elaborates the method, and the data presented in Breglia, F., Bouby, L., Wales, N. et al. Disentangling the origins of viticulture in the western Mediterranean. Sci Rep 13, 17284 (2023). https://doi.org/10.1038/s41598-023-44445-4

Are the claims properly placed in the context of the previous literature? Have the authors treated the literature fairly?

The reference list is incorrect. Page 2 [7] – Mangafa is not the source for the size and shape of wild vs cultivated pips. [13] – Dong et al. is the source for the two simultaneous domestication events 11,000 years ago in W Asia and Caucasus, which led to different routes and timing of dispersal and further introgressions of the primary cultivars. The reference [16] is dated to 2021, while [13] to 2023 – so the style of the sentence “However, the hypothesis of a second, independent, grape domestication event outside the original primary domestication center is still [sic! - reviewer] debated” - is confusing and misleading. ETC. It must be updated and fixed.

**Response**

Already answered above.

Page 2—Connection of the spread of viticulture with “emergence of complex societies”—please add more here for those who are not familiar with the development of societies in Italy. Add that in Italy, the first evidence of a hierarchical society was during the MB.

**Response**

Already answered above.

When citing only one single work in a sentence, such as “different research has employed pip outline analysis study grape subspecies [20] – the chosen reference should be a review, or please add “e.g.” before the single chosen work.

**Response**

Already answered above.

Do the data and analyses fully support the claims? If not, what other evidence is required?

The method applied in the research, Fourier transforms, is widely used for morphometric identification of plants, and grape pips. It is a strong method for machine learning identification. Unfortunately, the method cannot be used for manual identification, but this will be, hopefully, addressed later.

Nevertheless, it is important to add here in this paper a figure/ table to visualize the choice of the “six first harmonics, that were utilized here (although it is a continuation of the previously published method – ref 20 and 30. Accordingly, the data of the measurements – the raw data for statistics, should be presented. Now, it is presented in figure 4, where individual measurements are unclear. Next, the “48 EFT coefficients” – should be presented in the paper.

**Response**

Already answered above.

As for the results, means and covariances for the wild and domesticated types, as well, means and covariances for each period should be presented in the paper. This can be estimated from the training set. In this case, the data will help the readers to follow the authors and will allow us to evaluate the results, and to apply the results in future studies of other grape populations. Presenting the data will enlarge our understanding of the changes in the grape pip’s morphology with time and domestication.

Each method has its cautions and limitations, so it is critical to address this issue, and to discuss the pros and contra of applying the method on the results.

**Response**

Already answered above.

PLOS ONE encourages authors to publish detailed protocols and algorithms as supporting information online. Do any particular methods used in the manuscript warrant such treatment? If a protocol is already provided, for example for a randomized controlled trial, are there any important deviations from it? If so, have the authors explained adequately why the deviations occurred?

The paper does not present the raw data, so it is not possible to follow their protocol, nor to evaluate the data significance.

•	If the paper is considered unsuitable for publication in its present form, does the study itself show sufficient potential that the authors should be encouraged to resubmit a revised version?

The protocol must be presented (what are the vectors, and why they were chosen); and the data must be presented (mean, covar). Now it is unclear.

**Response**

Already answered above.
