# Supplementary material for: Tracing the emergence of domesticated grapevine in Italy
Source: PLoS One. 2025 Apr 23;20(4):e0321653. doi: 10.1371/journal.pone.0321653 (PMC12017526; doi:10.1371/journal.pone.0321653)
Supplement: Attachment — Submitted filename: review of revised manuscript.docx [file pone.0321653.s009.docx]

Review of revised manuscript: “Tracing the Emergence of Domesticated Grapevine in Italy”

The authors answered all the comments, including the raw and statistical data, and edited the reference list. The corrections made the paper ready for publication.

The paper presents important data that visualizes the non-linear evolution of wine domesticates in Italy. The raw data included now in the paper helps to follow the results and conclusions. I recommend the manuscript for publication in PLOS One.

I would suggest a minor change—in my opinion, a photo of grape pips from different stages of domestication would illustrate the process even better and significantly upgrade the visual appearance of the publication.

Sincerely,

Suembikya Frumin
