# Supplementary material for: Tracing the emergence of domesticated grapevine in Italy
Source: PLoS One. 2025 Apr 23;20(4):e0321653. doi: 10.1371/journal.pone.0321653 (PMC12017526; doi:10.1371/journal.pone.0321653)
Supplement: Attachment — Submitted filename: Response to Reviewers 2.docx [file pone.0321653.s010.docx]

**Reference** PONE-D-24-32533 *Tracing the Emergence of Domesticated Grapevine in Italy*

PLOS ONE

Dear editor,

We are grateful for the helpful feedback by the reviewers that helped us to improve the quality of the manuscript. We carefully responded to all points.

**Response to reviewer 2**

**Reviewers' comments:**

**Reviewer#2:**
**The paper presents important data that visualizes the non-linear evolution of wine domesticates in Italy. The raw data included now in the paper helps to follow the results and conclusions. I would suggest a minor change—in my opinion, a photo of grape pips from different stages of domestication would illustrate the process even better and significantly upgrade the visual appearance of the publication.**

Response

Thank you for your suggestion. We have included the images of the archaeological grape seeds within Figure 4. Each seed is positioned in line with the statistical results related to seed length.
